# Fatalism and metaphor in Confucianism: A qualitative study of barriers to genetic testing among first‐degree relatives of hereditary cancer patients from China

**DOI:** 10.1002/pon.6068

**Published:** 2022-11-19

**Authors:** Chaonan Jiang, Li Liu, Ye Wang, Liangzheng Wu, Wenxia Zhang, Xiaodan Wu

**Affiliations:** ^1^ State Key Laboratory of Oncology in South China Collaborative Innovation Center for Cancer Medicine Sun Yat‐sen University Cancer Center Guangzhou China; ^2^ The Cancer Center of Guangzhou Medical University Guangzhou China

**Keywords:** barriers, cancer, fatalism, genetic testing, hereditary cancer syndromes, metaphor, psycho‐oncology, relatives

## Abstract

**Objective:**

Despite the benefits, the rate of genetic testing among first‐degree relatives (FDRs; parents, children, and siblings) remains low, and the barriers to undergoing testing among FDRs in China are not clear. We explored the reasons why FDRs refused genetic testing.

**Methods:**

Semi‐structured face‐to‐face interviews were conducted with 22 patients and 27 FDRs. Participants were recruited at an urban tertiary hospital in Guangzhou, South China. We used qualitative content analysis to analyse the transcripts of audio recordings and identify major themes and subthemes.

**Results:**

Three major themes emerged related to FDRs' low rate of participation in genetic testing. First, there is cognitive distance from genetic testing/cancer and a lack of knowledge of preventive medicine that deepens the ‘fatalistic’ attitude towards cancer among FDRs, which leads to an enormous gap between their knowledge and understanding of genetic testing. Second, medical consultation is not valued in Confucianism, and the view of cancer as ‘bad news’ and the risk of cancer as a curse makes cancer a metaphor, which leads to exhausting arguments when persuading FDRs to undergo genetic testing. Third, physical distance from the hospital, loss of privacy, possible discrimination in many social activities and genetic testing as a source of stress and anxiety lead FDRs to fear the disruption of their daily lives.

**Conclusions:**

There are many barriers to genetic testing among the FDRs of hereditary cancer patients originating from the national social and cultural context. Healthcare professionals should develop interventions rooted in culture and promote cancer risk communication between hereditary cancer patients and FDRs.

## 1 BACKGROUND

Hereditary cancer is a cancer predisposition syndrome that confers a high lifetime risk of up to 40%∼80% of developing multiple malignancies, and it accounts for 10% of all cancers.^1^, ^2^ Hereditary breast‐ovarian cancer syndrome and hereditary colorectal cancer are the most common forms of hereditary cancer.^3^ The ability to identify genes that are associated with a high risk of colorectal cancer, breast cancer, ovarian cancer, and related cancer has advanced our understanding of hereditary cancer over the last decade.^4^, ^5^ First‐degree relatives (FDRs; parents, children, and siblings) of hereditary cancer patients have a 50% chance of carrying the patient's pathogenic genes.^6^ Genetic testing is available for mutations in several genes to predict the risk of most common hereditary cancers, such as breast and/or ovarian or colorectal cancer.^7^ FDRs who accept genetic testing can determine whether they are carriers of the genetic mutation, which can help carriers undertake strategies to reduce their risk of disease or facilitate early diagnosis and can help noncarriers avoid the costs of unnecessary interventions.^8^ Both outcomes yield benefits to individuals and society.

However, the rate of acceptance of genetic testing among FDRs in mutation‐positive families is very low. Previous research showed that 46%∼70% of members of families with hereditary cancer refused genetic testing in Italy, Australia, Canada and America.^7^, ^8^, ^9^ In China, there are few reports about genetic testing among the FDRs of hereditary cancer patients, while we previously discovered that 48% of the FDRs were informed about hereditary cancer in their families and only 10% of them completed genetic testing. These findings clearly indicate that there are still barriers hindering genetic testing for hereditary cancer. This phenomenon constitutes the core issue of this study: Why are there many practical barriers to genetic testing, which ultimately benefits the FDRs of hereditary cancer patients, and what specific barriers exist in the practice of genetic testing in China?

Previous research has explored why the FDRs of hereditary cancer patients refuse genetic testing from several perspectives.^10^, ^11^ Numerous studies have confirmed that genetic testing behaviours are affected by health insurance, education level, healthcare professional recommendations, health literacy and family supervision.^12^, ^13^ Perceived barriers to genetic testing were found to be the most important psychological factors. First, due to a lack of knowledge of disease susceptibility, severity and the significance of genetic testing and due to a lack of interest in preventive medicine, the acceptance of genetic testing among FDRs was found to be poor.^14^ Second, some people equated cancer with death,^15^ causing them to fear cancer and to disregard their risk of cancer. However, the existing studies emphasize individual direct experience while ignoring the social environment and the profound impact of cultural situations on individuals.^16^ Furthermore, most studies were undertaken in Western countries, including the United States and Europe,^17^, ^18^, ^19^ with few conducted in Asia,^20^ especially China.^21^ Confucianism has dominated Chinese culture and moral tradition for thousands of years. Influenced by Confucian culture, people believe that the occurrence of cancer is determined by fate and is not affected by one's own behaviour. Thus, they believe nothing can be done to reduce the occurrence of cancer. People feel powerless in the face of cancer and regard cancer treatment as a struggle against insurmountable odds. Even today, many Chinese people still adhere to traditional Confucian moral values. The cognition on cancer among Chinese people is different from that among people in Western countries due to the unique values held in China. In addition, cognition and research on hereditary cancer in China are still at an early exploratory stage. It is necessary to understand perceptions of genetic testing and the barriers among the FDRs of hereditary cancer patients in China. Therefore, in this study, we used a qualitative method to explore Confucian perspectives to identify factors that hinder genetic testing among a high‐risk population of FDRs of patients with hereditary cancer.

## 2 METHODS

### 2.1 Study design

This qualitative study utilized a grounded theory approach. We performed thematic analysis to explore and understand these complex issues. Hereditary cancer patients and their FDRs were recruited by purposive sampling between March 2021 and August 2021 at an urban tertiary hospital in Guangzhou, South China. This hospital was chosen because it has the largest known cohort of hereditary colorectal cancer patients and represents the highest level in the field of hereditary cancer in China. Forty‐nine semistructured and in‐depth interviews lasting from 37 to 58 min were performed face‐to‐face with the participants.

### 2.2 Participants

The inclusion criteria included (1) undergoing testing and genetic counselling for more than 1 year; (2) having at least one FDR (parents, siblings, or children) aged 18–70 years old that could be contacted; (3) diagnosed with hereditary cancer; (4) able to speak Mandarin; and (5) willing to participate in this study. Patients with medical or psychiatric diagnoses of cognitive or psychiatric disorders were excluded. In addition, we also recruited at least one FDR of each patient. The patients' parents, siblings, or children aged 18–70 years old were included, and those with mental illness or communication problems were excluded. To ensure the heterogeneity of the sample, we recruited not only FDRs who had never undergone genetic testing but also those who had hesitated for a long time before undergoing genetic testing. The FDRs' thoughts during the period of procrastination were considered valuable as indicators of barriers to genetic testing. Written informed consent was collected prior to the start of all interviews.

### 2.3 Data collection

An initial interview guide was developed on the basis of a literature review. After 3 interviews during the pilot phase and discussion with our team, the final interview guide included the following questions for the probands: ① What is your cognition about hereditary tumours? ② How do you think about the barriers to improving awareness of hereditary tumours in first‐degree relatives? ③ How do you think about the barriers to notifying first‐degree relatives of genetic testing? In addition, the questions for the FDRs included the following: ① How do you know about genetic testing? ② What is your understanding of the population's high risk of cancer? ③ How do you experience genetic testing? Two researchers conducted the interviews in Mandarin after establishing relationships with participants prior to the study commencement. We used maximum variation sampling to recruit a wide range of diverse participants.^22^ No new information emerged in the last three interviews, indicating that data saturation was reached, and no additional interviews were conducted.

### 2.4 Data analysis

We analysed the data through a process including open coding, the creation of categories, and abstraction. The interviews were audio recorded and transcribed anonymously in Chinese within 24 h after each interview. Two researchers independently and continuously reviewed and coded the transcriptions of the recordings and developed a draft coding framework through line‐by‐line coding. After a careful comparison and discussion of divergences between the two researchers, the open coding was finalised. Then, we used qualitative content analysis to generate themes and subthemes. Typical quotes from participants were chosen to support each theme and answer the research questions. The participants' names were replaced with numbers (e.g., P1) to ensure confidentiality.

## 3 RESULTS

Ultimately, 50 potential participants, including 22 patients and 28 FDRs, were approached; one FDR declined to be interviewed due to time constraints (Table 1). There is no specific difference found in different types of hereditary cancer. Younger participants were found to more easily accept genetic testing than older participants while they were busy coping with jobs in daily life, which influenced their adherence to genetic testing.

**TABLE 1:** Description of the study participants

Characteristic	Patients' information (no. %)	FDRs' information (no. %)
Age, mean (SD), y	39.21 ± 10.94 (26–58)	
Sex		
Female	13 (59.1)	18 (66.7)
Male	9 (40.9)	9 (33.3)
Type of hereditary cancer		
Hereditary breast‐ovarian cancer	8 (36.4)	10 (37.0)
Lynch syndrome	10 (45.4)	12 (44.5)
Familial adenomatous polyposis	4 (18.2)	5 (18.5)
Relation to the patient		
Parent	/	9 (33.3)
Sibling	/	12 (44.5)
Child	/	6 (22.2)
Educational level		
Did not complete high school	8 (36.4)	9 (33.3)
Completed high school and <4 years college	10 (45.4)	11 (40.7)
College graduate or postgraduate degrees	4 (18.2)	7 (26.0)
Received genetic testing or colonoscopy in the last 5 years		
No	/	23 (85.2)
Yes	/	4 (14.8)

Three major themes emerged to describe the barriers to undergoing genetic testing.

### 3.1 ‘Fatalistic’ attitude towards cancer

Recent developments in modern medicine have clarified the links between cancer and genetics, which can be utilized to definitively establish a diagnosis for hereditary cancer patients. However, at the same time, these links further strengthen FDRs' ‘fatalistic’ attitudes towards cancer occurrence because of their cognitive distance from cancer, which results in barriers to genetic testing.

#### 3.1.1 Cognitive distance from genetic testing and cancer

Many FDRs regard cancer as terminal illness and death and often avoid discussing related topics. The medical experience of these patients around their FDRs informed their understanding of cancer and influenced their decisions to obtain genetic testing.

*My younger sister died of colorectal cancer at the age of 21. My mother was diagnosed with endometrial cancer 4 years ago, and now she is well. I hear that one can be cured if he or she is diagnosed at an older age. Younger people with cancer are all incurable. And I think colorectal cancer is terrible; maybe endometrial cancer is easier to treat. The difference between my sister and my mother was predestined; what way do I have again? (F5, patient's daughter, aged 32).*

#### 3.1.2 Lack of knowledge of preventive medicine

The patients and their FDRs had developed their own concepts of health and preventive medicine based on various indicators, including no signs and symptoms of cancer in routine physical examinations. The FDRs' understanding of diseases and prevention was complex, which made them reluctant to accept genetic testing and cancer screening. They did not realize that routine physical examination could not completely replace genetic testing and targeted screening. As one participant said.

*I think that normal annual physical examination results meant good health. There is no need for deliberate genetic testing. My work unit organizes a physical examination every year, and if a physical examination can detect any problem, why go to so much trouble to do a special examination? No, I have never done genetic testing or colonoscopy. (F8, patient's daughter, aged 25)*.

#### 3.1.3 ‘Fatalistic’ attitude towards cancer occurrence and prognosis

A ‘fatalistic’ attitude towards cancer caused people to distance themselves from genetic testing. Some FDRs believed that the risk of cancer was not influenced by their own behaviour and that everything was predestined and could not be changed.

*I resolutely refuse to do genetic testing, as this kind of thing depends on fate. If you are destined to have cancer, you will never change this fate! (F7, patient's brother, aged 37*).

Some FDRs believed that genetic testing would simply prove that they were destined to have cancer, which would have no practical function. As one participant said.

*You have told me this disease is genetically related, so getting genetic testing would just prove this known fact. Can you change my bad genes? It is impossible, so genetic testing make no sense. (F14, patient's son, aged 29*).

#### 3.1.4 Enormous gap between knowledge and understanding owing to ‘fatalistic’ attitudes

The patients and FDRs likely found it difficult to understand the doctor's message because of the overuse of medical jargon in communicating and their fatalism accompanied by insufficient medical knowledge.

*My doctors all were nice and spent a lot of time talking with me. But what they explained made me confused. I don't understand why it is helpful to do genetic testing. They told me my father carried the same genes with me in all probability, but he has not yet suffered from cancer; he is 62 years old now. I had cancer at age 32. (P7, patient, aged 35)*.

### 3.2 Cancer as a metaphor in confucianism

In China, under the influence of Confucian culture, people associate death with pain and fear, and ‘cancer’ has a special metaphorical meaning similar to terminal disease and death. It is taboo to talk about cancer, which leads to barriers in informing and persuading FDRs to accept genetic testing.

#### 3.2.1 Risk of cancer as a curse

People usually use terms such as ‘this disease’ or ‘inflammation’ instead of ‘cancer’ in their communication. Advising someone to undergo genetic testing was seen as a curse.

*Suffering from ‘inflammation’ is not a good thing, not a glorious thing. When I told this to my old brother, he didn't want to listen to me. I tried to persuade him to do the genetic testing, and he was very angry with me. Since then, he has refused to answer the phone when I call. (P18, patient, aged 54*).

#### 3.2.2 Arguments exhausted

Telling relatives straightforwardly that they were at risk of hereditary cancer was unwelcome. Therefore, the patients lacked the skills to persuade their FDRs to undergo genetic testing.

*Instead of saying that cancer is prevalent in our family, somebody would misunderstand what the other said. My uncle firmly believed three of our relatives didn't have cancer, but I know from the hospital that they indeed had cancer. I felt helpless about how to communicate with them. (P19, patient, aged 31*).

#### 3.2.3 Medical consultation not valued in China

Medical consultation is not taken seriously in Chinese culture. Instead, people place emphasis on therapeutic intervention. Many doctors may not have the time and energy to communicate and inform individuals because of their heavy workloads, which aggravates the decrease in consultations.

*My doctor has the final say in making the decision if I have the disease. When you get cancer, just listen to your doctor for treatment. It makes no sense to do consulting in advance. The doctors have no time to listen to too much. (F13, patient's brother, aged 40*).

### 3.3 Fear of the disruption of daily life

The physical distance between patients and the hospital and the time and transportation cost to perform genetic testing increased the barriers to genetic testing. In addition, the possible negative outcomes arising from genetic testing could directly disrupt the participants' lives and immerse them in fear. They refused to be labelled negatively based on their genetic susceptibility to disease.

#### 3.3.1 Physical distance from the hospital

Some FDRs lived far from the hospital, introducing problems such as time coordination and transportation to reach the site of genetic testing, which would interrupt their work and life. As one of our participants said.

*It is difficult to receive genetic testing independently for me. Many hospitals cannot perform this testing; only here can they do it, but I live so far from your hospital. I often required assistance from my children when I go out, and my children are very busy working. (F4, patient's brother, aged 55*).

#### 3.3.2 Loss of privacy and possible discrimination in many social activities

Genetic testing and the possibility of being identified as gene carriers would lead FDRs to experience a loss of privacy and possible discrimination in many social activities, such as competing for job positions and acquiring health insurance. Therefore, they were more likely to choose to maintain their current lives than to be exposed to unpredictable testing results.

*Many companies eliminate genetic diseases from commercial health insurance coverage. My commercial health insurance would lapse if I was identified as a susceptibility gene carrier. (F7, patient's brother, aged 37*).

*My son and daughter would be discriminated against in their career development and looking for a marriage partner as a susceptibility gene carrier, so I can't let it happen. (P18, patient, aged 54*).

#### 3.3.3 Source of stress and anxiety

The participants described that if being a gene carrier was confirmed by testing, they would live with the stress of having cancer. Like this participant said.

*Cancer is incurable and inevitable, which makes me feel stressed, as I just think about it. Why should I identify that I will have cancer? That will only make me depressed all day for the rest of my life. As long as I do not do the testing, I feel in good health. (F13, patient's brother, aged 40*).

#### 3.3.4 Maintain one's daily life prior to genetic testing

The daily lives of some FDRs were occupied by complex work, caring for their parents, parenting, and other activities or events. The possible negative outcomes of genetic testing could lead their daily lives to be completely controlled by the hospital, which would make them feel powerless.

*I'm busy every day. I should look after the little baby for my son on his days. On the weekend, I go to the park every morning to dance, then buy vegetables to cook, sometimes go to class in the afternoon, and often have activities in the evening. I am now fulfilled every day and living a healthy life without having to go for testing. (F1, patients' sister, aged 53*).

## 4 DISCUSSION

### 4.1 Summary of findings

The FDRs of hereditary cancer patients are a high‐risk population for cancer.^3^ Performing genetic testing is ‘a step forward’ in the prevention of hereditary cancer in the sense that high‐risk individuals can be precisely identified, and cancer surveillance can be limited to gene carriers, thus avoiding unnecessary early investigations in many individuals. At the same time, gene carriers should be identified to begin early cancer screening to detect and treat precancerous lesions in a timely manner to avoid progression to advanced cancer.^23^ Despite the benefit, a large fraction of high‐risk FDRs in families with hereditary cancer do not undergo genetic tests. Some studies^13^ found that among the examined subjects, the large majority of gene carriers followed the recommendations and were willing to undergo colonoscopic surveillance. Therefore, it is vital to increase the rate of genetic testing. This study found that there were three barriers to genetic testing among the FDRs of patients with hereditary cancer. We specifically elaborated from the three perspectives.

First, the gap between fatalism about cancer and preventive medicine is difficult to bridge. More than 50% of people in China believe that cancer is an incurable disease. These people have also developed the view that having cancer means terminal illness and death. As genetic testing is a newly developed medical detection technology, many people in China do not understand its significance. Previous studies have found that cancer patients, their FDRs and some medical experts have limited awareness of hereditary cancer.^13^, ^24^ All of these factors further strengthen people's fatalistic attitudes and cognition away from prevention and make individuals deny the significance and value of genetic testing. Cognitive interventions based on the Confucian cultural context may be designed to change the status quo. The awareness of hereditary cancer in clinical staff should be strengthened. Meanwhile, healthcare providers should help FDRs develop the correct concept of health and preventive medicine that heredity cancer is not incurable or unavoidable and understand the significance of tertiary prevention by sharing scientific evidence about hereditary cancer. Moreover, the role of advanced genetic nursing should be emphasized in the future and to develop detailed intervention plans.

The present study found that FDRs' genetic testing behaviour should be considered in light of Confucian culture and the unique Chinese cultural context. People associate cancer with death and fear, and it is taboo to talk about cancer under the influence of Confucian culture, with the result that cancer is metaphorically referred to as ‘this/that disease’. Human ethics is an extremely important aspect of Chinese traditional culture, which contains the five cardinal relationships defined in feudal China, and questions concerning life and death are very serious. Assuming that FDRs would develop a serious disease is very offensive and unpleasant. Therefore, informing and persuading FDRs to accept genetic testing is unwelcome; this is especially true for FDRs with whom one has no opportunity to communicate face‐to‐face, as family harmony is perceived as the most vital social value in Confucian culture. Many FDRs are unwilling to accept the fact that they are at high risk of cancer and reject related information. Straightforward discussion is not accepted, and the use of metaphor is not valued, which causes conflict in communication about risk for hereditary cancer among patients and FDRs. Many Chinese people still adhere to traditional Confucian moral values today. Traditional Chinese society is a hierarchical society with patriarchal clan systems. Following this model, Chinese people trust information from relatives/friends and online sources more than information from doctors, who are ‘strangers’. Encouraging hereditary cancer patients to talk about the risk of cancer that runs in the family and the possibilities for prevention and early diagnosis is a part of the popularization of knowledge. The knowledge level of an individual will have an impact on the concept of fatalism. Standardizing popular science knowledge about cancer diagnosis, treatment and prognosis on the internet and spreading accurate and positive knowledge through health education websites can reduce people's notions of cancer fatalism. Direct telephone calls from cancer genetic counselors to the FDRs of hereditary cancer patients may be a good approach. Narrative nursing is considered to be an effective mode of psychological intervention by encouraging people to share their cancer‐related experiences. Positive spiritual belief was used to change people's negative concepts about cancer.

Finally, individuals' thinking and existing lifestyles tend to be maintained without change.^19^ A possible unforeseen event arising from genetic testing limits their willingness to complete testing. The possible loss of privacy and discrimination in many social activities due to genetic risk may lead to unfair treatment in daily life.^25^ To promote genetic testing, medical professionals should debunk the myths and misconceptions associated with cancer. Ethics education should be strengthened in clinical practice, and information technology should be used to strictly prevent the leakage of patients' genetic information and protect patients' privacy. It is necessary to establish a better social security system to ensure patients' medical expenses and relieve their financial concerns. Taking measures to better educate patients, the institution of specialized units entirely devoted to inherited tumours and further collaboration with mass media should be performed to remove relevant barriers to genetic testing in families with hereditary cancer. Consequently, we would be able to provide adequate protection against cancer development in these families.

### 4.2 Study limitations

The hereditary cancer patients and FDRs were recruited from only one tertiary care hospital in an urban area and did not focus on highly populated but underdeveloped area regions, hospitals or communities. Second, considering ethical issues, the views of some FDRs who were unaware of the diagnosis of hereditary cancer of the index case were not approached. In addition, future studies should investigate the role of culture in the process and mechanism of interacting with family communication, perception and beliefs.

### 4.3 Clinical implications

To the best of our knowledge, this is the first study to identify Chinese perceptions about genetic testing among a high‐risk population of FDRs of patients with hereditary cancer from China. The results of this study can contribute to the evidence‐based literature on barriers to genetic testing and ultimately to improve the rate of genetic testing among the FDRs of patients with all hereditary cancers. It provides a methodological reference for the sociocognitive and behavioural study of the high‐risk population for hereditary cancer. Healthcare providers should take some interventions rooted in culture to change the way people think about cancer, especially develop the correct concept of cancer and preventive medicine, and understand the significance of tertiary prevention. Narrative nursing is considered to be an effective mode of psychological intervention by encouraging hereditary cancer patients to share their cancer‐related experiences, such as the risk of cancer that runs in the family and the possibilities for prevention and early diagnosis.

## 5 CONCLUSIONS

We found that the FDRs of hereditary cancer patients in China generally refused to participate in genetic testing despite being at high risk for hereditary cancer. This can be attributed to three major barriers: 1) a ‘fatalistic’ attitude towards cancer occurrence; 2) cancer as a metaphor in Confucianism; and 3) fear of the disruption of daily life. Moreover, technology, especially unfamiliar genetic testing, should be rooted in the national social and cultural context to increase the testing rate in China to achieve the objective of helping molecular medicine save lives. It is suggested that in genetic testing practice, we should pay attention to the influence of the overall sociocultural atmosphere on each individual and family. The development of interventions should also be rooted in culture, and we should promote communication between hereditary cancer patients and FDRs. FDRs' cognitive distance from cancer and their ‘fatalistic’ attitude towards cancer should be changed. Some countermeasures and policies should be developed to protect the individuals affected by susceptibility genes from discrimination.

## AUTHOR CONTRIBUTIONS

Xiaodan Wu, Chaonan Jiang, Li Liu conceived and designed the study; Chaonan Jiang, Xiaodan Wu carried out the research interviews; Li Liu, Liangzheng Wu, Wenxia Zhang, Ye Wang analysed the transcripts; Xiaodan Wu, Li Liu, Ye Wang drafted a report of the findings, and all authors commented on the analysis; Xiaodan Wu, Li Liu, Chaonan Jiang drafted this manuscript, and all authors commented on revised versions. All authors read and approved the final manuscript.

## CONFLICT OF INTEREST

The authors declare no conflicts of interest.

## ETHICS STATEMENT

Ethical approval was obtained from the ethics committee of Sun Yat‐sen University Cancer Center (approval number: B2021‐373‐01).

## Data Availability

The data presented in this study are available on request from the corresponding author. The data are not publicly available due to confidentiality requirements and the personal nature of the research topic and questions discussed.
